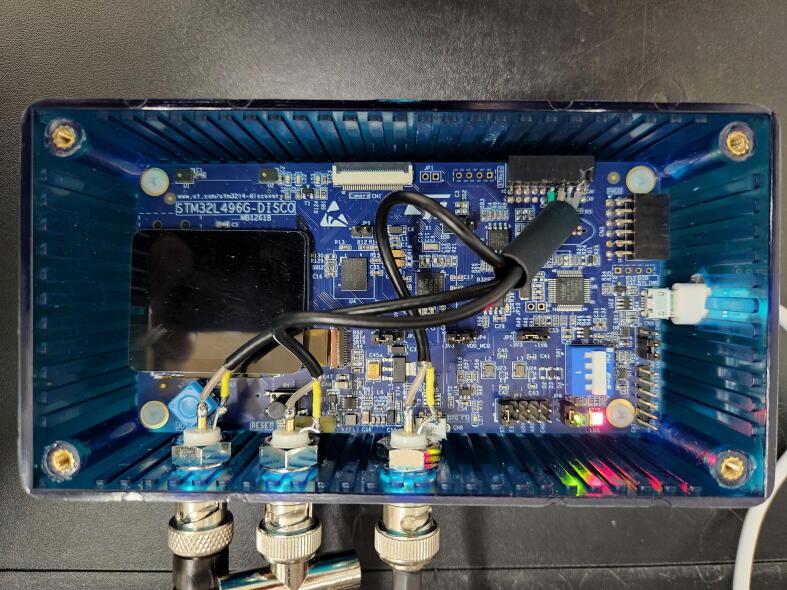# Synchronization of scanning probe and pixelated sensor for image-guided diffraction microscopy

**DOI:** 10.1016/j.ohx.2023.e00431

**Published:** 2023-05-24

**Authors:** Shahar Seifer, Michael Elbaum

**Affiliations:** Chemical and Biological Physics, Weizmann Institute of Science, Rehovot, Israel

**Keywords:** Scanning transmission electron microscopy, 4D-STEM, Electron tomography, Compressed sensing, Tilt series

## Abstract

A 4-dimensional modality of a scanning transmission electron microscope (4D-STEM) acquires diffraction images formed by a coherent and focused electron beam scanning the specimen. Newly developed ultrafast detectors offer a possibility to acquire high throughput diffraction patterns at each pixel of the scan, enabling rapid tilt series acquisition for 4D-STEM tomography. Here we present a solution to the problem of synchronizing the electron probe scan with the diffraction image acquisition, and demonstrate on a fast hybrid-pixel detector camera (ARINA, DECTRIS). Image-guided tracking and autofocus corrections are handled by the freely-available microscope-control software SerialEM, in conjunction with a high angle annular dark field (HAADF) image acquired simultaneously. The open source SavvyScan system offers a versatile set of scanning patterns, operated by commercially available multi-channel acquisition and signal generator computer cards (Spectrum Instrumentation GmbH). Images are recorded only within a sub-region of the total field, so as to avoid spurious data collection during flyback and/or acceleration periods in the scan. Hence, the trigger of the fast camera follows selected pulses from the scan generator clock gated according to the chosen scan pattern. Software and protocol are provided for gating the trigger pulses via a microcontroller (ST Microelectronics ARM Cortex). We demonstrate the system on a standard replica grating and by diffraction imaging of a ferritin specimen.

## Graphical abstract

**Figure:**
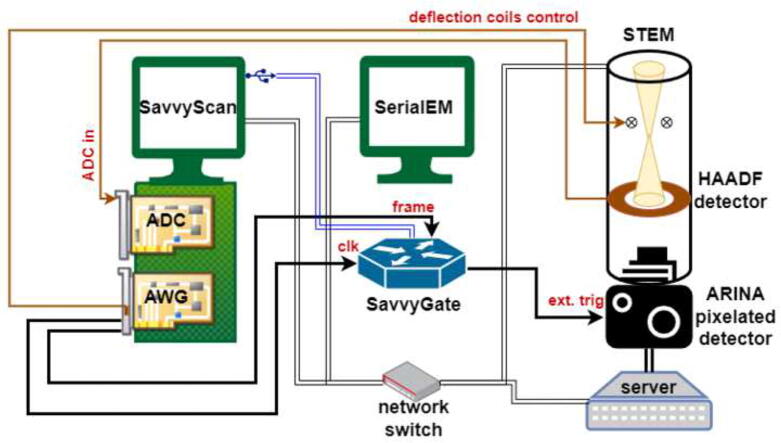

**Specifications table**

**Table:** 

Hardware name	*SavvyGate*
Subject area	• Engineering and materials science • Biological sciences (e.g., microbiology and biochemistry)
Hardware type	• Imaging tools • Structural biology and materials science
Closest commercial analog	*Point Electronic DISS 6; JEOL STEM; Gatan STEMx; NanoMEGAS Digistar; TVIPS Universal Scan Generator; Direct Electron DE-FREESCAN (these do not support tilt series acquisition in 4D-STEM)*
Open source license	*CC BY 4.0 (Creative Common Attribution 4.0 International)(note: the code depends on firmware libraries that are open source under BSD-3 license, which means that a product may not be endorsed or promoted by the name of STMicroelectronics.)*
Cost of hardware	*100 USD*
Source file repository	https://doi.org/10.5281/zenodo.7673922

## Hardware in context

Scanning transmission electron microscopy (STEM) uses a focused beam as a probe to measure electron scattering in transmission through a specimen. The scattering wave projects a convergent-beam electron diffraction (CBED) pattern in the far field [1]. Classical STEM techniques such as high angle annular dark field (HAADF) or bright field (BF) STEM use area detectors to record the scattered intensity by essentially integrating the electron flux over specific regions of the diffraction pattern. The emerging method of 4D-STEM uses a pixelated camera to record a 2D image of the CBED for each position, i.e., pixel, of the electron probe in real space. Integrations over areas specified *a posteriori* create virtual detectors, from which the real space images (scan images) display a variety of contrast aspects. The projected electrical potential induced by the atomic nuclei in the specimen affects the phase of the electron wave. A phase image may be obtained using a quadrant detector, real or virtual, placed at the diffraction plane. This already offers a possibility to compensate for defocus aberration and reveal depth contrast from a single scan [2]. More precise phase reconstruction can be made by the methods of integrated center of mass (iCOM) imaging [3] or Ptychography [4], which require recording the full CBED pattern. Acquiring the full 2d diffraction information enables, at least in theory, to apply reconstruction schemes such as inverse multislice reconstruction [5], multislice electron tomography [6] and atomic model iterative reconstruction [7], for enhanced resolution in the final images. Finally, specimens are susceptible to damage by the electron beam. The approach of compressed sensing suggests to reduce electron exposure with minimal loss in information via sparse sampling in the real space acquisition, i.e., by skipping probe positions [8]. The combined approaches are now offered in a single a system, which acquires 2d diffraction information, guides the microscope during specimen tilting, and manages the scanning pattern. The system is based on available components and common interfaces of a standard transmission electron microscope, and is supported by open-source software.

The goal in this work is to synchronize the collection of diffraction images with the pixel clock of a STEM scan generator at the relevant pixel time on the order of 10 µs, while maintaining the ability to guide the microscope based on real-time image feedback during acquisition. It offers a first of a kind solution for a tilt-series acquisition in 4D-STEM. We build here upon a previously reported flexible scan system, called SavvyScan [2], which combines an arbitrary wave generator (AWG) to control the scan coils of the microscope, with an eight-channel analog to digital converter (ADC) acquisition card for signal recording from multiple electron detectors. In the present implementation, we acquire a single channel of data from a conventional high angle annular dark-field (HAADF) detector, while simultaneously triggering the clock of a fast electron-counting hybrid camera (ARINA, DECTRIS AG, Switzerland). In particular, we describe the software and hardware design to collect only useful data during the scans, i.e., to avoid distortions and overdose, and to acquire the signals simultaneously.

The synchronization of scan position, analog channel acquisition, and pixelated detector recording is more challenging than it might appear. First of all, a typical raster scan for STEM includes a fly-back time where the beam returns or accelerates and the image information is distorted, due to inductive inertia in the scan coils [2]. Previous designs select the information during post-processing [9], but here we require selective recording in advance because the overload of storage and processing time is significant. Second, we require a real-time feedback for purposes of focusing and navigation across the grid. This is based on images provided by the HAADF detector (note, however, that in some ultra-high resolution applications a careful aberration corrections in HAADF may not be optimized for bright field images [10]). The HAADF signal is collected by an analog to digital converter (ADC) acquisition card that integrates up to 64 samples per pixel at a fast oversampling rate in order to reduce electronic noise; therefore, the same pulses cannot serve for both ADC and camera. As a solution, a master sampling clock from the ADC serves as a reference for the clock of the arbitrary wavefunction generator (AWG) that controls the scan coils of the microscope. The clock pulses of the AWG card are then used to trigger the pixelated detector, but the trigger is enabled only at useful locations of the probe. Specifically, sampling is skipped during the flyback and acceleration phases of the raster scan, thereby avoiding distortions in the image. The same scheme of editing the trigger enable list may be employed for compressive sensing applications, which propose to reproduce a full image from a small fraction of sampled data points. In order to reduce electron exposure in practice, the sub-sampling must be coupled in addition with a fast shutter to blank the beam while the non-recorded points are visited.

**Fig. 1:**
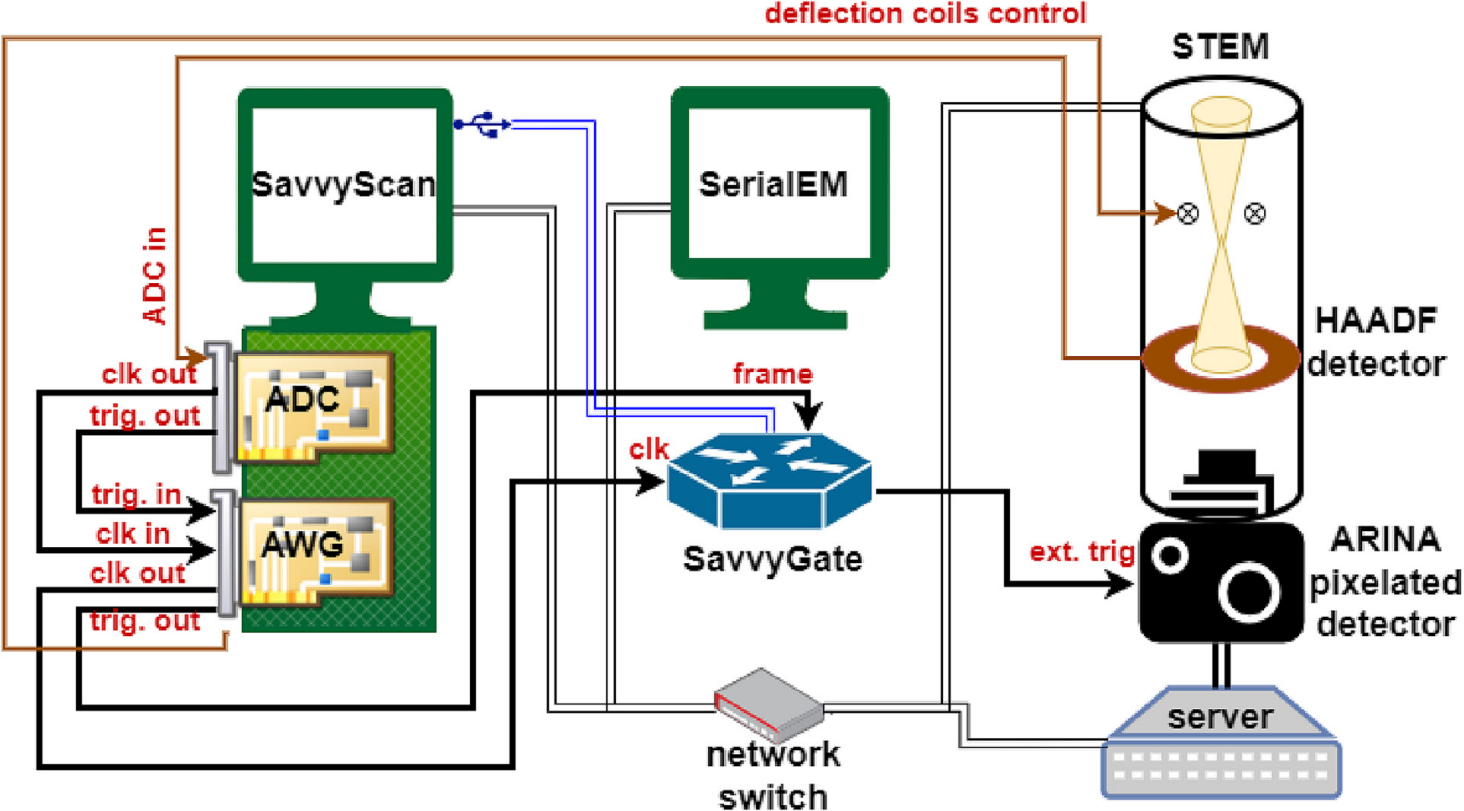
Connection scheme of the SavvyScan system integrated with ARINA pixelated detector.

The synchronization system is called SavvyGate. Fig. 1 provides a connections scheme of the flexible scanning SavvyScan system. The hybrid detector camera is installed on the transmission electron microscope (Tecani F20, Thermo Fisher Scientific) and stores a stream of images (up to 120,000 images per second at 96 × 96 pixels, and at least 20,000 per second at 192x192 pixels) on a dedicated server, in real time, in compressed HDF5 file format. The server is attached to a local Ethernet network, which includes the SavvyScan computer that sets the acquisition mode and initiates full data recording, and a separate computer that controls the microscope optics and mechanical stage via the SerialEM software framework and scripting interface to the microscope [11]. The HAADF image sourced from the ADC card (Spectrum Instruments M2p.5923-x4) is fed back to SerialEM for purposes of navigation, tracking, focus, etc. The AWG card (Spectrum Instruments M2p.6541-x4) generates the scanX,Y signals. The usual synchronization between these cards is based on a component called STAR-HUB; however, it is outside specification for clock rate slower than 128 kHz, so the synchronization is set as shown in the diagram. The ADC trigger, which indicates the start of an acquisition on command from SerialEM, is fed to the trigger input of the AWG. The output trigger from the AWG is a frame HIGH signal during the run of the scan pattern, and is fed to the SavvyGate microcontroller unit (MCU). In order to allow both synchronization and oversampling, the ADC clock rate must be an integer multiple of the AWG clock rate. To maintain stability, the output clock from the ADC is fed as a reference to a phase-locked loop of the AWG clock. The output clock signal of the AWG is then fed to SavvyGate, which generates selective pulses of 3 µs width according to a sequence table delivered from the SavvyScan computer via a Universal Serial Bus (USB) port.

The SavvyScan system functions as an ad hoc camera server for SerialEM software and provides an immediate image, which is necessary for the user as well as for automated image guided tasks in SerialEM such as tracking alignment, autofocus, and eucentric height alignment. The HAADF detector is often a favorable option to generate rapid image guidance while the diffraction images are collected for off-line processing. The HAADF signal is also useful for image alignments in tomography based on strongly-scattering fiducials such as gold nanoparticles. However, in specimens thicker than the elastic scattering mean free path some details are lost in the HAADF, and it is necessary to use bright field image for microscope guidance. Accordingly, the system should ideally return a real-time image signal such as iCOM or virtual bright field computed on the fly from the pixelated detector measurements. Such capability is recently offered in the LiberTEM-live project [12], which aims further to achieve resolution enhancement based on ptychography on the fly [13]. SavvyScan version 2b can mediate between LiberTEM-live output and SerialEM to facilitate real-time tilt-series acquisition and montage mapping based on 4D-STEM data stream. Nevertheless, HAADF is still required for the quick intermediate scans and the SavvyGate hardware supports synchronization with the scanning probe. A further route of development is the extension to unconventional scan modes such as spirals, sliding circles, or Hilbert patterns. In such cases the real-time generation of a HAADF image for SerialEM will always be available without complication. SavvyScan is already compensating for the distortions due to inductive inertia in the scan coils for the analog signal [2], and similar compensations would have to be applied to the camera-generated images.

## Hardware description

**Fig. 2:**
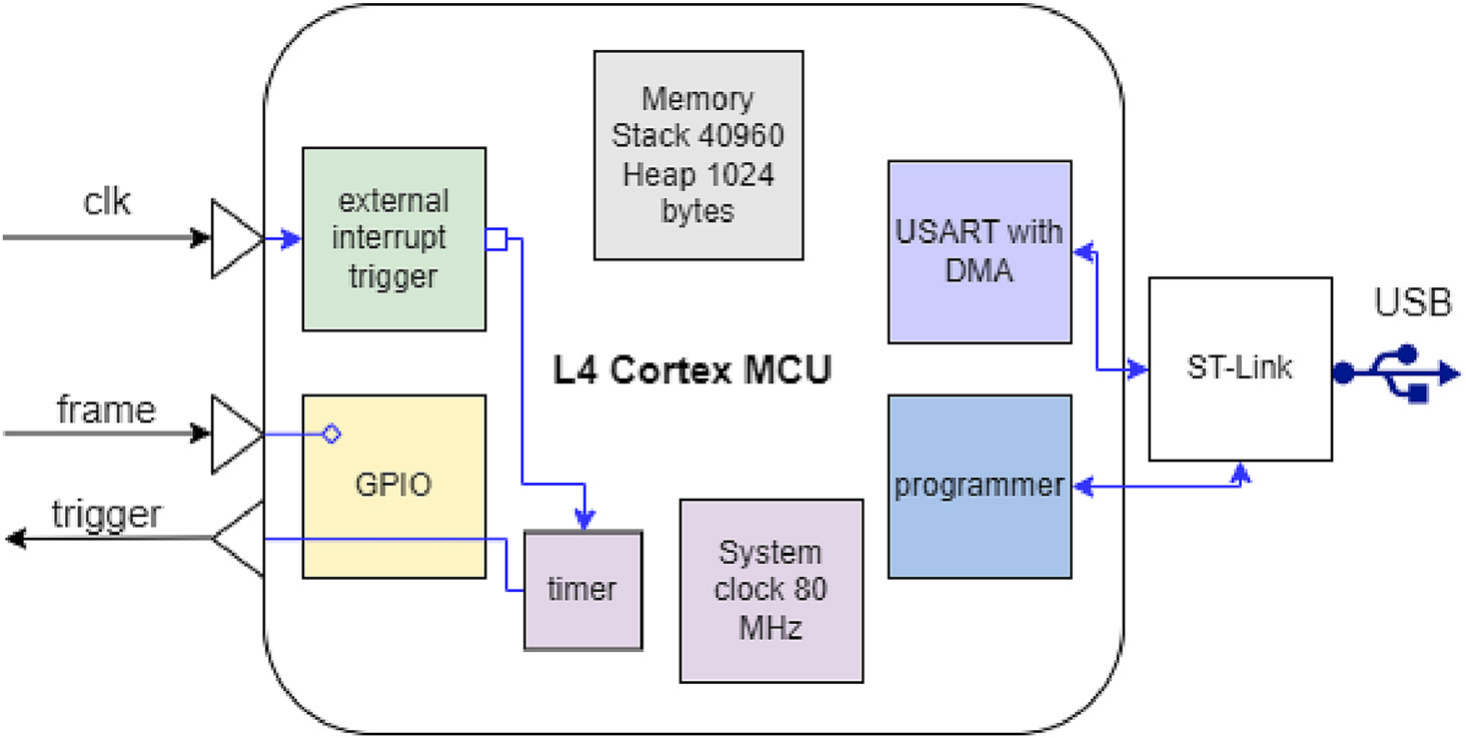
Block diagram of SavvyGate.

The purpose of SavvyGate is to send trigger pulses to the camera. The functional block diagram is shown in Fig. 2. Programming and communication are provided by the USB port, which also supplies the power. A sequence table in memory must be loaded before each scan using a direct memory access (DMA) streaming from the Universal Serial Asynchronous Receiver Transmitter module connected to ST-Link USB module. In the context of STEM the sequence table is determined by the SavvyScan system. The sequence starts once the sequence table is loaded and the frame state is high. The rising edge of the clock input activates an interrupt, which upon a condition defined by the frame signal and position in the sequence table the trigger is followed by generation of a trigger pulse to the camera.

The sequence table contains 20 pages of 2500 words in a little endian byte order. The last word in each page serves to indicate if the last packet of information is received, so only the necessary number of pages are transferred. In all other 16-bit words the most significant bit specifies if to transmit (1) or skip (0) a train of pulses. The count of the pulse train is provided in the least significant 15 bits of the word. For example, a raster scan of 1024X1024 pixels requires 2048 words. Each line of scan requires one word for the skip train (meaning the number of pulses to skip, which are 400 pulses including flyback from the previous line and the travel of the probe outside the range of interest), and one word for the transmit train of 1024 pulses. Using 8 pages of 2500 words we can acquire 8 k scan images. Pulse trains longer than 32,767 clocks may be achieved by concatenation as needed. In more sophisticated patterns that involve compressed sensing, the number of trains may be larger, and it would be possible to control the beam illumination with a fast shutter using SavvyGate to reduce the electron dose.

## Design files summary

**Table:** 

**Design file name**	**File type**	**Open source license**	**Location of the file**
*SavvyGate project*	Firmware source files (project in Atollic TrueStudio C compiler and STM32CubeIDE)	CC BY 4.0 (contribution) BSD-3 (dependencies)	https://doi.org/10.5281/zenodo.7673922
*example_send_pattern.cpp* *Example_operate_cards.cpp*	PC code example	CC BY 4.0	https://doi.org/10.5281/zenodo.7673922
*SavvyScan project, version 2*	Visual Studio C++ project	GPL-3	https://github.com/Pr4Et/SavvyScan

## Bill of materials summary

**Table:** 

**Designator**	**Component**	**Number**	**Cost per unit -currency**	**Total cost – currency**	**Source of materials**
MCU board based on STM32L496 MCU	*32L496GDISCOVERY* *ST Micro*	1	70 USD	70 USD	https://www.st.com/en/evaluation-tools/32l496gdiscovery.html#sample-buy
BNC connector bulkhead mount	CONBNC004 Amphenol	3	2.7 USD	8.1 USD	
Enclosure	1591DTBU Hammond Manuf.	1	13.9 USD	13.9 USD	

## Build instructions

• Set jumpers for the STM32L496G discovery board according to default positions. The CN5 USB connector is used as a multi-purpose power source, debugger and communication port.
• Connect BNC socket “clock in” to STMod+ socket pin 18 (PC2 pin of the MCU).
• Connect BNC socket “frame in” to STMod+ socket pin 17 (PC7 pin of the MCU).
• Connect BNC socket “trigger out” to STMod+ socket pin 19 (PB2 pin of the MCU).
• Connect BNC grounds to STMod+ socket pin 16 (GND).
• Prepare the enclosure with holes for the BNC sockets and USB cable (see prototype image in the supplementary information).
• Install ST-Link driver (*https://www.st.com/en/development-tools/stsw-link009.html*) on a Windows personal computer (PC) and connect the discovery board using a USB cable.
• Install the free Atollic TrueSTUDIO (v.9.3.0) on a PC. The ST-Link firmware of the board can be upgraded using Help, Tools, ST-Link Upgrade. Load the SavvyGate project, Build and Debug (the latter burns the program on the STM32L496AG MCU). The compiler is set for using Thumb2 with floating point in hardware implementation FPv4-SP-D16.
• Set the COM port properties (using Device Manager in Windows) to 115,200 bits per second, databits 8, parity none, stop bit 1, flow control none.
• Adding functionalities may be easily implemented using STM32CubeIDE software with the supplied ioc project file.

## Operation instructions

• Connect the discovery board ST-Link socket to the PC (the virtual COM port may be operated either in parallel to debugging the board, or in standalone mode after debugging). Check that the firmware is correctly installed by sending 5000 bytes of zeros to the virtual COM port and receiving OK message back (see program example_send_pattern.cpp).
• Connect the SavvyGate frame input to a signal source (for example, trigger output of Spectrum AWG card [x1 socket], set according to program Example_operate_cards.cpp).
• Connect the SavvyGate clock input to a signal source (for example, clock output of Spectrum AWG card [x0 socket], set according to program Example_operate_cards.cpp).
• To test sending pattern to SavvyGate follow the code example_send_pattern.cpp, which only requires setting the tables locx[j], locy[j] according to a scanning pattern, and updating the COM port number.
• To test synchronization with Spectrum ADC and AWG cards follow the code Example_operate_cards.cpp, or install the complete SavvyScan project with version 2 updates, available in GitHub [14].

## Validation and characterization

**Fig. 3:**
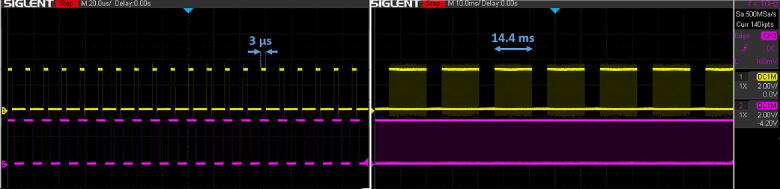
Trigger pulses (3 µs wide) generated in SavvyGate (yellow) vs. the scanner clock signal from the ADC (magenta). Note the delay of 1 µs between the rising edges. The zoom-out timeline on the right shows a 14.4 ms pulse train sequence in a typical raster scan. (For interpretation of the references to color in this figure legend, the reader is referred to the web version of this article.)

Fig. 3 shows a typical clock signal generated by the AWG card in a raster scan (magenta trace), together with the SavvyGate output of 3 µs pulses (yellow trace) revealing a propagation delay of 1 µs after the rising edge of the input clock. This implies that the firmware supports up to 250 kHz pixel clock rate, which is not the final capability of the hardware. The right part of Fig. 3 reveals several pulse trains that correspond to a similar number of scan lines. The gaps between the pulse trains correspond to periods when the probe is traveling outside the region of interest and accelerates toward a constant velocity of travel. In SerialEM we have set the time per pixel at “20 µs/pix”, which is in terms of the overall scan time divided by 1024X1024 useful pixels. The overall time of our raster scan pattern includes 29.3% part overhead for the flyback and acceleration periods, so the actual duration of the probe over a pixel is 14.1 µs. The field of view is exposed to electron damage only during 70% of the overall scan time. We allowed for a setting time of 3 µs (for demonstration) in the ARINA detector before the trigger, so the integration time in ARINA was set to 10 µs, in which the electrons are counted. The setting time can be reduced further to 0.5 µs, which in addition to 1 µs propagation delay implies a minimum 1.5 µs “dead time” in which a point of interest in the specimen is exposed to radiation but electrons are not counted.

**Fig. 4:**
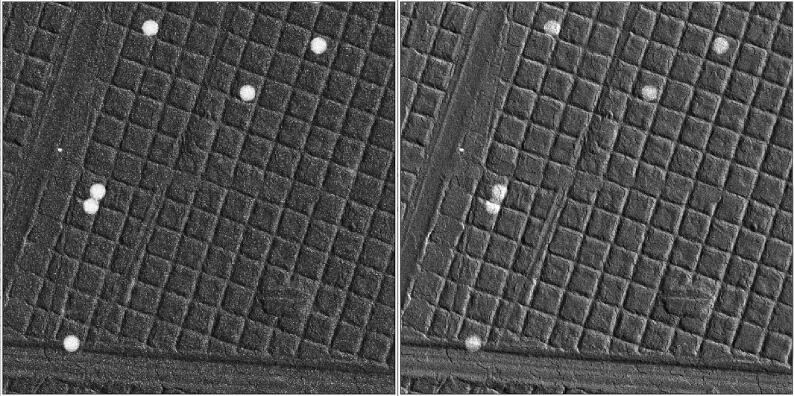
(left) vDF image from 4D-STEM data acquired by ARINA camera, (right) HAADF scan image of a replica grating with 0.26 µm latex spheres.

The experiment was performed on a scanning transmission electron microscope (Tecnai T20-F, Thermo Fisher Scientific) set to acceleration voltage of 200 kV (De Broglie wavelength of 2.5 pm) in microprobe mode (convergence angle of 0.8 mrad) and about 5pA electron current. In order to test synchronization between the HAADF detector (Fischione 3000) and the ARINA camera (DECTRIS), a virtual dark field image (vDF) is computed from the diffraction pattern recorded on the latter. The intensity of each pixel is determined by the sum of all electron counts falling between radii of 12 and 48 pixels on the diffraction images (measured from the center of the diffraction disc). A common term used to specify the magnification of the diffraction pattern is the camera length, in analogy to an equivalent camera obscura setup. At an effective camera length setting of 390 mm, the corresponding scattering range included in the vDF is between 0.26 and 1.03 Å^−1^ (6.4 and 25.6 mrad), whereas the HAADF covers scattering range between 1.1 and 4.4 Å^−1^. Also, the analog HAADF signal is oversampled by 64 digital samples per pixel by the SavvyScan ADC for noise reduction, whereas the ARINA camera is counting electrons. In Fig. 4 we show the scan images acquired simultaneously for a specimen of replica grating with latex spheres from Electron Microscopy Sciences (EMS cat. #80055). The vDF image is shifted to the left by 8 pixels because of delay in the AWG Spectrum card with relation to its own frame signal. This has little implications for using the HAADF signal as guiding image. HAADF images are also useful for acquiring the relative alignment shifts of the tilt series. However, the HAADF images should be aligned with the vDF images before combining the detector inputs for extended diffraction information.

**Fig. 5:**
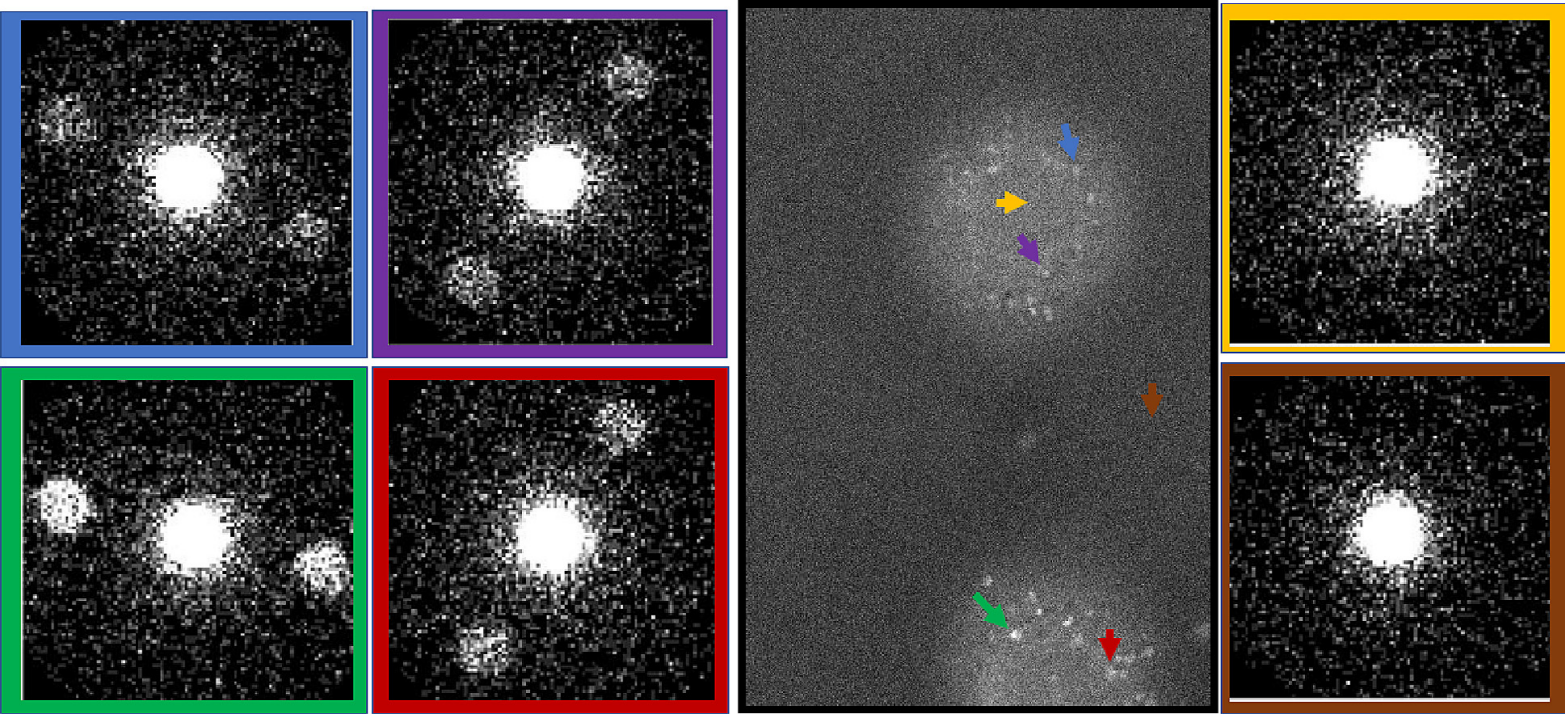
4D-STEM demonstration in Ferritin aggregates (300 nm in size). Bragg patterns in CBED appear at certain locations indicated in the vDF scan by colored arrows.

The 4D-STEM modality is demonstrated on aggregates of ferritin found on a purchased dry specimen (Ferritin on a grid, EMS cat #80042). The size of the aggregates is about 300 nm while the known diameter of a single ferritin molecule is 12 nm. In Fig. 5 we show vDF scan image and various CBED diffraction images averaged over squares of 5X5 scan pixels at the locations indicated by the colored arrows, which correspond to the frame color of the CBED pattern. At some probe locations the Bragg reflections are dominant, exhibiting a twofold symmetry that has been reported previously in polymers [15]. The twofold diffraction pattern could arise from a structure with translational symmetry (2_R_) as well as other point groups [16]. The effective camera length was 1940 mm so the scattering vector magnitude at the Bragg disc locations is 0.16 Å^−1^, which conforms to a lamellar structure with periodic interlayer spacing of 6.2 Å. The Bragg condition is met where the 30 Å wide beam is almost parallel to the scattering planes, thus the orientation of the discs reveal the normal direction to the lamellar structure. A possible explanation for the emergence of scattering planes is a periodic variation in the iron content within the ferritin molecules [17]. Using a much larger probe size (and camera length) would reveal the global crystalline order among ferritin molecules [18]; however, the current system is unique in mapping the variation in the internal structure within every pixel in the scan.

**Fig. 6:**
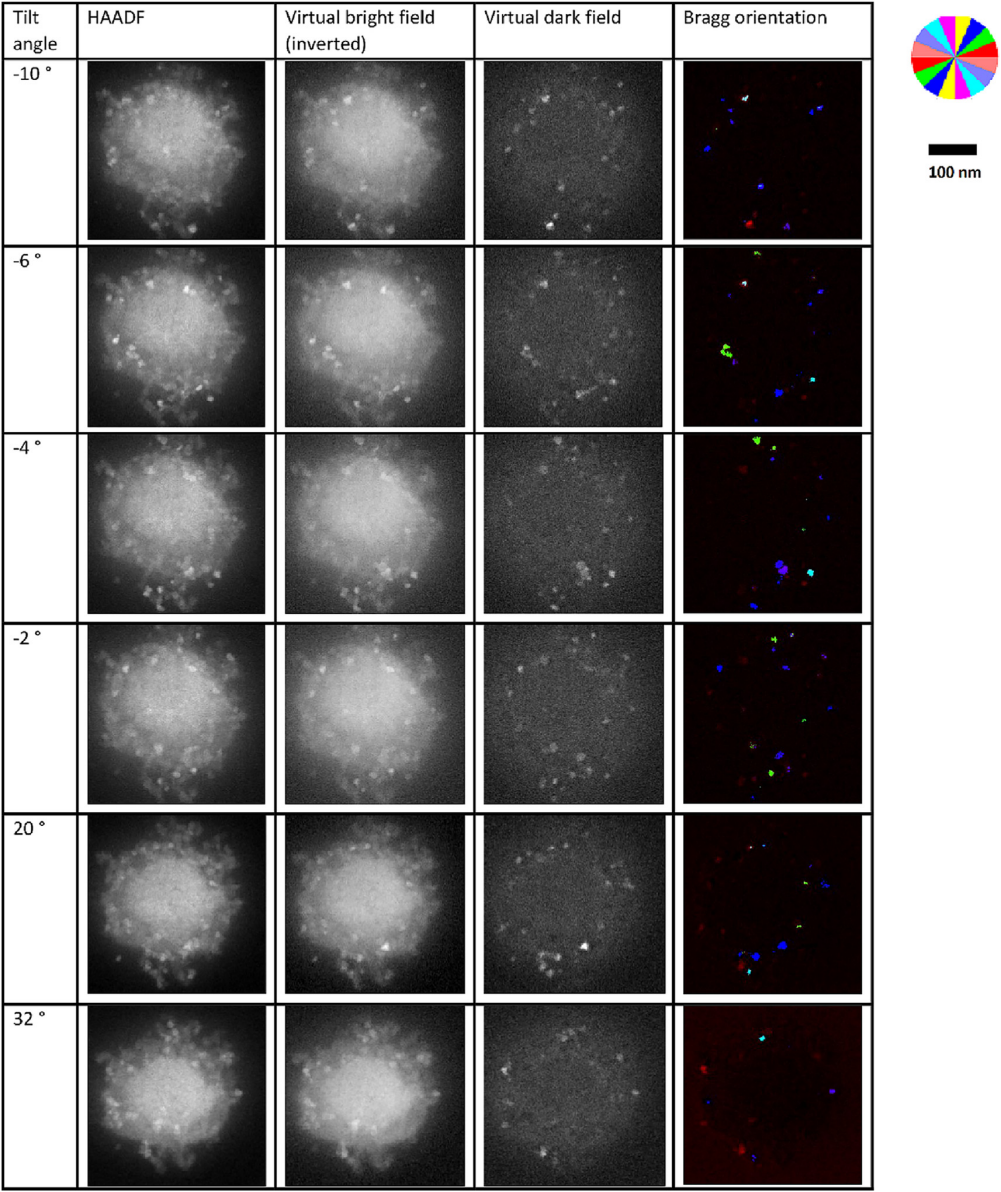
Ferritin aggregate at different tilt angles demonstrating 4D-STEM information in a tilt series.

Crystalline grains diffract at specific tilt angles so it is interesting to rotate the specimen. Starting from version 2 of SavvyScan [14] the tilt series acquisition with ARINA detector is completely automated. The ferritin specimen was tilted between − 36⁰ and 36⁰ at steps of 2⁰ and scanned using the tilt series acquisition function in SerialEM [11]. A “trial” scan preceded each “record” scan to align the microscope position; whereas the SavvyScan system operated the electron camera only during “record” scans based on the scan settings reported to the system by the serialEM plugin. SerialEM was able to track and correct the scanning location as for its usual application to tomography. The HDF5 files recorded by the DECTRIS server of 40 GB total size were processed according to virtual detector segments to generate MRC files. The tilt series MRC stacks were aligned according to a rigid body rotation model by ClusterAlign [19]. The region of interest was selected at the same location for all tilt views. The results are shown in Fig. 6 for the HAADF scans in comparison to the vDF and virtual bright field images at several tilt views. The dominant shining features may conform to crystalline grains of a few ferritin molecules, within which the lamellar structure happens to meet the Bragg reflection condition (i.e., beam almost parallel to the scattering planes). For those molecules, we were able to map the orientation of the scattering planes by the displayed color code.

In summary, the ongoing trend in electron microscopy to study of beam sensitive materials, including cryogenic biological specimens [4], [20], largely motivates the development of dose-efficient methods for STEM. 4D-STEM is especially attractive for such because it can employ essentially every scattered electron for generation of useful contrast, but until recently the approach was severely limited by long image acquisition times. The system presented here addresses the significant technical problem of synchronizing the scan with the diffraction image acquisition at the relevant rates on the order of 100 kHz. Building on the previously-reported scan generator, which was designed for acquisition from a segmented diode STEM detector (Opal, El-Mul Technologies, Israel) and integrated with the microscope control platform SerialEM [2], the system provides a complete image acquisition solution for 4D STEM.

## CRediT authorship contribution statement

**Shahar Seifer:** Conceptualization, Methodology, Software, Investigation, Data curation, Writing – original draft. **Michael Elbaum:** Conceptualization, Investigation, Writing – review & editing, Supervision.

## Declaration of Competing Interest

The authors declare that they have no known competing financial interests or personal relationships that could have appeared to influence the work reported in this paper.

## Figures and Tables

**Figure:**